# Meaning and Late-Life Vulnerability: A Philosophical Practice-Based Approach

**DOI:** 10.1093/geroni/igaa057.3069

**Published:** 2020-12-16

**Authors:** Hanne Laceulle

**Affiliations:** University of Humanistic Studies, Utrecht, Netherlands

## Abstract

The quest for meaning constitutes one of the fundamental human existential motivations. At first glance, late life vulnerability appears to pose a threat to meaningfulness. Sources of meaning associated with certain activities or relations may seem less accessible once vulnerabilities, caused by social losses or diminishing health, start to dominate people’s daily life experience. On the other hand, some theorists have argued that it is particularly in the confrontation with our human vulnerability that the need for meaningfulness is most urgently felt. This paper presents a philosophical-conceptual exploration of meaning as a dynamic, context-dependent social practice. Contrasting theoretical approaches that analyse meaningfulness and/or meaninglessness in terms of the presence or absence of certain components or qualities, a practice-based approach enhances our sensivitity for concrete ways in which people manage to find or create meaning in situations of vulnerability, thereby integrating this vulnerability in their life narratives and sense of self.

